# Beyond the Norm: A Report of a Rare Case of Sodium Channel 8 Alpha (SCN8A) Gene-Related Epilepsy Unveiled in a Nine-Year-Old Child

**DOI:** 10.7759/cureus.59775

**Published:** 2024-05-07

**Authors:** Sri Sita Naga Sai Priya K, Keta Vagha, Ashish Varma, Chaitanya Kumar Javvaji, Krupa Bhanushali, Aashita Malik, Anuja Handargule

**Affiliations:** 1 Pediatrics, Jawaharlal Nehru Medical College, Datta Meghe Institute of Higher Education and Research, Wardha, IND

**Keywords:** antiepileptic drugs, nav1.6 sodium channels, genetic testing, refractory epilepsy, epileptic encephalopathy (dee), - scn8a mutation

## Abstract

Sodium channel 8 alpha (SCN8A) mutations encompass a spectrum of epilepsy phenotypes with diverse clinical manifestations, posing diagnostic challenges. We present a case of a nine-year-old male with SCN8A gene-associated developmental and epileptic encephalopathies (DEEs), characterized by generalized tonic-clonic seizures (GTCS) since infancy. Despite treatment with multiple antiepileptic drugs (AEDs), including phenytoin, valproate, levetiracetam, carbamazepine, and clobazam, seizure control remained elusive, prompting genetic testing. Whole exome sequencing confirmed a heterozygous mutation (p.Phe210Ser) in SCN8A exon 6, indicative of DEE-13. Functional studies revealed a gain-of-function mechanism in SCN8A variants, resulting in heightened ion channel activity and altered voltage dependence of activation. Despite treatment adjustments, the patient's seizures persisted until topiramate was introduced, offering partial relief. SCN8A, encoding Nav1.6 sodium channels, modulates neuronal excitability, with mutations leading to increased persistent currents and hyperexcitability. Early seizure onset and developmental delays are hallmarks of SCN8A-related DEE. This case highlights the significance of genetic testing in refractory epilepsy management, guiding personalized treatment strategies. Sodium channel blockers like phenytoin and carbamazepine are often first-line therapies, while topiramate presents as a potential adjunctive option in SCN8A-related DEE. Overall, this case underscores the diagnostic and therapeutic complexities of managing SCN8A-related epileptic encephalopathy, emphasizing the importance of long-term monitoring and personalized treatment approaches for optimizing outcomes in refractory epilepsy.

## Introduction

Sodium channel 8 alpha (SCN8A) encodes Nav1.6, a voltage-gated sodium channel predominantly found in excitatory neurons, with particularly dense expression at the axon initial segment and the nodes of Ranvier [1]. Pathogenic mutations in the SCN8A gene are linked to various epilepsy phenotypes, spanning from benign familial infantile seizures (BFIS) in select families to severe developmental and epileptic encephalopathies (DEEs) that manifest early in life [2].

The majority of patients diagnosed with SCN8A-associated DEEs typically exhibit a severe phenotype. This includes the early onset of seizures, often proving challenging to manage with conventional treatments. Additionally, these individuals commonly experience severe intellectual disability, motor impairments, and unfortunately, a relatively high mortality rate [3].

Functional studies of selected variants associated with DEEs have unveiled a gain-of-function mechanism as the primary pathogenic driver [4]. This gain of function arises from heightened activity of the ion channel, attributed to increased persistent sodium currents, shifts toward hyperpolarization in the voltage dependence of activation, or impaired inactivation of the channel current [5].

This case report highlights the diagnostic journey and therapeutic interventions in a nine-year-old child presenting with intractable epilepsy. Despite treatment with various antiepileptic medications, the patient's seizures remained refractory, underscoring the complexity of managing epilepsy in pediatric populations. 

## Case presentation

A nine-year-old male was brought to our tertiary care rural hospital in central India with complaints of generalized tonic-clonic seizures (GTCS) lasting over 10 hours. He is the second child of non-consanguineous parents, weighing 3.5 kg at birth without complications.

The patient's convulsive episodes commenced at one and a half years of age, initially manifesting as simple febrile seizures. By the age of three, he began experiencing multiple GTCS episodes without associated fever. Following hospitalization, he was discharged on a regimen of phenytoin for a two-year duration. Despite this, he continued to experience one to two GTCS convulsive episodes monthly associated with postictal drowsiness lasting for 15-20 minutes.

At five years of age, valproate and levetiracetam were introduced in addition to phenytoin. At eight years old, a new convulsive episode occurred during a febrile episode, prompting hospitalization. Carbamazepine and clobazam were added to the treatment regimen, alongside risperidone due to hyperactive behavior.

During the latest admission, the patient received loading doses of injectable phenytoin, valproate, and levetiracetam, while maintaining previous medications. After stabilization, a neurologist recommended discontinuing carbamazepine and initiating lacosamide. During the latest admission, the patient received loading doses of injectable phenytoin, valproate, and levetiracetam, while maintaining previous medications. After stabilization, the pediatric neurologist recommended discontinuing carbamazepine and initiating lacosamide. A sleep-awake electroencephalogram (EEG) revealed ictogenic discharges in the right centro-temporal region. Given persistent hyperactive behavior, risperidone dosage was increased, and phenytoin was gradually tapered and discontinued. The patient was discharged with a plan to gradually wean off levetiracetam while maintaining valproate, risperidone, lacosamide, and clobazam.

**Figure 1:**
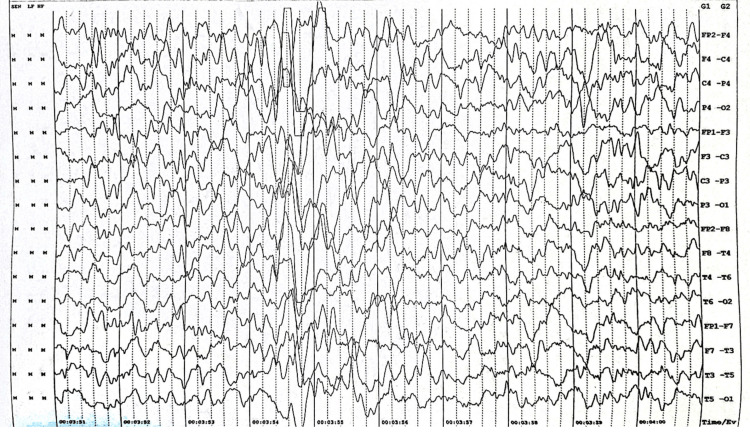
The electroencephalograph shows generalized intermittent sharp and high amplitude waves in the center-temporal-parietal region, reflecting ongoing ictogenic discharges.

Post-seizure, the patient exhibited no postictal drowsiness, and physical examination revealed no facial dysmorphisms, with normal ophthalmic evaluations and vitals within the expected range. The pulse rate was 102 beats per minute, respiratory rate of 26 cycles per minute, saturation of 96 was measured over the right index finger, and blood pressure of 102/68 mmHg (50th to 90th centile). Anthropometric measurements indicated typical growth, while neurological examination unveiled hypertonia in all limbs with extensor plantar reflexes. Laboratory tests showed random blood glucose of 99 mg/dL, sodium 142 mEq/L, potassium 4.3 mEq/L, calcium 9.6 mg/dL, magnesium 1.8 mg/dL, phosphorous 3.9 mg/dL, a hemoglobin level of 10.6 g/dL, a total leukocyte count of 11,400/cu mm, and a platelet count of 422,000/cu mm. The EEG findings revealed repetitive sharp and high-amplitude waves in the right centrotemporal region, suggestive of ictogenic discharges as shown in Figure 1.

**Figure 2:**
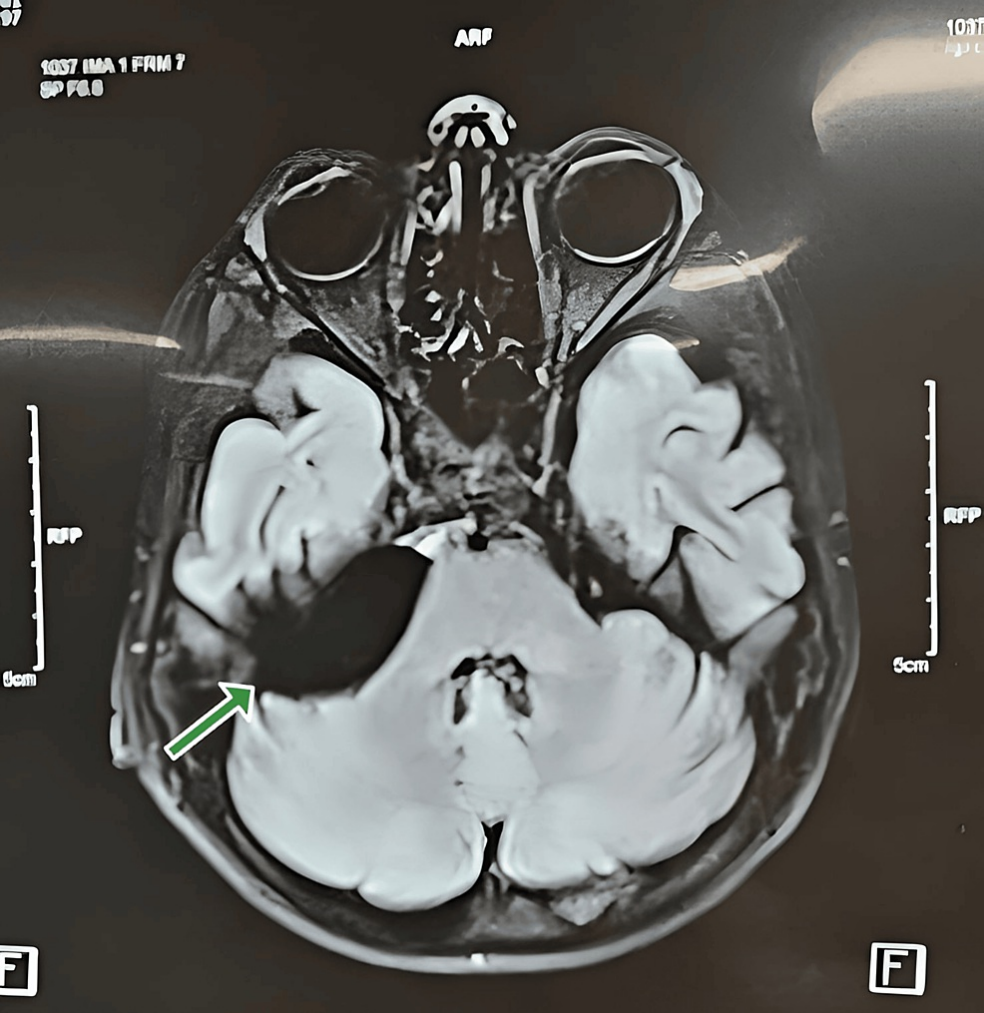
Magnetic resonance imaging through the posterior fossa demonstrated a large right-sided extra-axial cerebrospinal fluid intensity mass lesion. There is a significant mass effect on the adjacent cerebellar tissue.

An MRI disclosed an arachnoid cyst at the cerebellopontine angle on the right side as depicted in Figure 2.

After genetic testing, a heterozygous mutation was identified in the SCN8A gene at exon 6 (p.Phe210Ser), indicating DEE-13.

The patient presented again with recurrent episodes of GTCS lasting for 20 hours with 10 to 12 episodes, necessitating stabilization with injectable valproate and levetiracetam. Due to the persistence of continuous simple partial convulsions, lacosamide was discontinued, and topiramate was initiated, resulting in partial seizure control. Subsequently, the patient was discharged following improvement.

Ultimately, the patient was discharged while maintaining a medication regimen consisting of valproate, levetiracetam, topiramate, clobazam, and risperidone.

## Discussion

SCN8A is extensively distributed in both the central and peripheral nervous systems. It is located on chromosome 12q13 and encodes the alpha 8 subunit of the neuronal voltage-gated sodium channel, Nav1.6 [6]. This channel forms a complex with beta subunits to regulate current conductance. Nav1.6 comprises four transmembrane domains (DI-DIV), each consisting of six segments (S1-S6). The S1-S4 segments form the voltage-sensing domain, while the S5-S6 segments, along with the P-loop between them, constitute the pore-forming domain [7].

Within each domain, there are critical amino acid residues responsible for sodium ion selectivity, voltage sensing, and channel gating. Nav1.6 channels swiftly open upon depolarization, enabling sodium influx crucial for initiating action potentials and facilitating neuronal communication. Following this, channels undergo inactivation, crucial for halting action potentials and preventing sustained firing [8].

Mutations in SCN8A result in premature channel opening, impaired inactivation, and an augmented persistent current [6].

Complete inactivation of SCN8A decreases repetitive firing, resurgent current, and persistent current in various brain cells such as cerebellar Purkinje cells, prefrontal cortical pyramidal cells, and hippocampal cornu ammonis area 1 (CA1) cells [9]. These alterations result in a reduction in the frequency of action potentials, likely contributing to intellectual disability.

Missense mutations in SCN8A, constituting approximately 1% of cases of epileptic encephalopathy (EE), are linked to a diverse range of epilepsy phenotypes. These mutations are known to manifest in early infantile EE, characterized by a broad spectrum of seizures including focal, generalized, and epileptic spasms [10].

The median age of seizure onset typically occurs around five months of age, ranging from postnatal day 1 to 18 months. Patients often present with multiple seizure types. Additionally, the majority of individuals with SCN8A mutations experience varying degrees of global developmental delay, ranging from mild to severe [11].

The case of a nine-year-old male child with a history of GTCS and developmental delays underscores the challenges in managing refractory epilepsy and the importance of genetic testing. Initially presenting with simple febrile convulsions at one and a half years old, the patient's seizure frequency escalated, necessitating multiple antiepileptic medications. Despite treatment adjustments over the years, including phenytoin, valproate, levetiracetam, carbamazepine, and clobazam, achieving seizure control remained elusive.

Our patient had been prescribed multiple antiepileptic drugs before admission, with topiramate added as a final resort due to the persistence of continuous simple partial seizures. While there was some partial response observed with topiramate, many patients require maintenance on multiple medications to achieve partial seizure control [12]. Both phenytoin and carbamazepine function as sodium channel blockers, with carbamazepine having approximately three times lower affinity for sodium channels compared to phenytoin [13]. In our case, phenytoin was discontinued due to its side effects.

This case highlights the diagnostic journey in identifying SCN8A-related EE, necessitating multiple antiepileptic medications and eventual genetic testing. Long-term management and monitoring are essential in optimizing outcomes for patients with SCN8A mutations and refractory epilepsy.

## Conclusions

In conclusion, the identification of a heterozygous mutation in the SCN8A gene, specifically the p.Phe210Ser variant, in our patient provides valuable insights into the underlying etiology of their refractory epilepsy and developmental delays. This mutation is indicative of DEE-13, highlighting the importance of genetic testing in cases of intractable epilepsy. The clinical course of our patient, characterized by the need for multiple antiepileptic medications and eventual partial response to topiramate, underscores the challenges in managing SCN8A-related disorders and the importance of personalized treatment approaches guided by genetic findings.

## References

[1] O'Brien JE, Drews VL, Jones JM, Dugas JC, Barres BA, Meisler MH (2012). Rbfox proteins regulate alternative splicing of neuronal sodium channel SCN8A. Mol Cell Neurosci.

[2] Veeramah KR, O'Brien JE, Meisler MH (2012). De novo pathogenic SCN8A mutation identified by whole-genome sequencing of a family quartet affected by infantile epileptic encephalopathy and SUDEP. Am J Hum Genet.

[3] Johannesen KM, Gardella E, Scheffer I (2018). Early mortality in SCN8A-related epilepsies. Epilepsy Res.

[4] Blanchard MG, Willemsen MH, Walker JB (2015). De novo gain-of-function and loss-of-function mutations of SCN8A in patients with intellectual disabilities and epilepsy. J Med Genet.

[5] Wagnon JL, Barker BS, Hounshell JA (2016). Pathogenic mechanism of recurrent mutations of SCN8A in epileptic encephalopathy. Ann Clin Transl Neurol.

[6] Meisler MH (2019). SCN8A encephalopathy: mechanisms and models. Epilepsia.

[7] Zybura A, Hudmon A, Cummins TR (2021). Distinctive properties and powerful neuromodulation of Nav1.6 sodium channels regulates neuronal excitability. Cells.

[8] Arisaka A, Nakashima M, Kumada S (2021). Association of early-onset epileptic encephalopathy with involuntary movements - case series and literature review. Epilepsy Behav Rep.

[9] Gardella E, Marini C, Trivisano M (2018). The phenotype of SCN8A developmental and epileptic encephalopathy. Neurology.

[10] Wagnon JL, Meisler MH (2015). Recurrent and non-recurrent mutations of SCN8A in epileptic encephalopathy. Front Neurol.

[11] Guo QB, Zhan L, Xu HY, Gao ZB, Zheng YM (2022). SCN8A epileptic encephalopathy mutations display a gain-of-function phenotype and divergent sensitivity to antiepileptic drugs. Acta Pharmacol Sin.

[12] Boerma RS, Braun KP, van den Broek MP (2016). Remarkable phenytoin sensitivity in 4 children with SCN8A-related epilepsy: a molecular neuropharmacological approach. Neurotherapeutics.

[13] Kuo CC, Chen RS, Lu L, Chen RC (1997). Carbamazepine inhibition of neuronal Na+ currents: quantitative distinction from phenytoin and possible therapeutic implications. Mol Pharmacol.

